# ﻿*Phragmothecacentinelensis* (Malvaceae, Malvoideae or Matisioideae), a newly-discovered, critically-endangered canopy tree species from a cloud forest in Pacific Ecuador

**DOI:** 10.3897/phytokeys.254.143106

**Published:** 2025-03-21

**Authors:** Juan Ernesto Guevara-Andino, Dawson M. White, Nigel C. A. Pitman, Juan-Carlos Cerón, Andrea Fernández, Daniel Navas-Muñoz, William S. Alverson

**Affiliations:** 1 Grupo de Investigación en Ecología y Evolución en los Trópicos-EETrop- Universidad de las Américas, Quito 170124, Ecuador Universidad de las Américas Quito Ecuador; 2 Harvard University Herbaria, 22 Divinity Ave., Cambridge, MA 02476, USA Harvard University Herbaria Cambridge United States of America; 3 Collections, Conservation & Research, Field Museum of Natural History, 1400 S. Lake Shore Dr., Chicago, IL 60605, USA Collections, Conservation & Research, Field Museum of Natural History Chicago United States of America; 4 Herbario Nacional del Ecuador (QCNE), Instituto Nacional de Biodiversidad, Av. Río Coca E6-115 e Isla Fernandina, Quito, Ecuador Instituto Nacional de Biodiversidad Quito Ecuador; 5 Herbario QCA, Escuela de Ciencias Biológicas, Pontificia Universidad Católica del Ecuador, Av. 12 de Octubre 1076 y Roca, Apartado 17-01-2184, Quito, Ecuador Pontificia Universidad Católica del Ecuador Quito Ecuador; 6 WIS Herbarium, University of Wisconsin–Madison, 430 Lincoln Dr., Madison WI 53706, USA University of Wisconsin–Madison Madison United States of America

**Keywords:** Biological collections, Centinela, deforestation, endemism, extinction, Malvatheca clade, Matisieae, Matisioideae, Colecciones biológicas, Centinela, deforestación, endemismo, extinción, clado Malvatheca, Matisieae, Matisioideae

## Abstract

During floristic inventories in remnant cloud forests of the Centinela Ridge of the Chocó Region of Ecuador, where less than 7 km^2^ of forest patches remain across an area of approximately 500 km^2^, we recently collected a new species in the genus *Phragmotheca* Cuatrec. We describe and illustrate this new species and contrast its morphology with known congeneric species. Due to its small range, threatened habitat and active targeting by loggers, this species is assessed as Endangered under IUCN Criterion B1B2ab(I,ii,iii,v).

## Abstract (es)

Durante inventarios florísticos en los remanentes de bosques nublados de la cordillera Centinela, en la región del Chocó en Ecuador, donde menos de 7 km^2^ de parches de bosque permanecen en un área de aproximadamente 500 km^2^, recientemente recolectamos una nueva especie del género *Phragmotheca* Cuatrec. Describimos e ilustramos esta nueva especie y contrastamos su morfología con las especies congéneres conocidas. Debido a su distribución restringida, hábitat amenazado y la explotación activa por parte de madereros, esta especie se evalúa como En Peligro según el Criterio B1B2ab(i,ii,iii,v) de la UICN.

## ﻿Introduction

The genus *Phragmotheca* Cuatrec. (Malvaceae, Malvoideae or Matisioideae) comprises 11 known species (Table 1): four in lowland Amazonian forests of Colombia, Ecuador and Peru; two in the Andean highlands of Colombia; and five in low- and mid-elevation Pacific-slope forests of Colombia and eastern Panama (Alverson 1991; Fernández-Alonso 1996; Fernández-Alonso & Jaramillo-Mejía 1999, Fernández-Alonso et al. 2017). Species are generally large canopy trees (to 50 m) with the exception of *P.lemniscata* Fern.Alonso, which is a treelet in the Chocó Region of Colombia. *Phragmotheca* is closely related to *Matisia* Bonpl. and *Quararibea* Aubl. The three genera consistently form a clade in phylogenetic analyses (/Matisieae in Baum et al. (1998, 2004)) and have been placed in the Matisieae K.Schum. (Schumann 1895; Reveal 2012) within the Malvoideae (sensu Baum et al. (1998, 2004); the /Malvoideae clade of Alverson et al. (1999)). A new revision of ranked names within Malvaceae (Colli-Silva et al. 2025) proposes that the /Matisieae clade be renamed Matisioideae, but one of us [WSA] considers this change to be unjustified at present (see Appendix 1). *Phragmotheca* differs from the other two genera by its conspicuous internal partitioning of staminal thecae into foveae or septa (cf. fig. 1 in Alverson (1991) and figs 4, 8, 9 and 12 in Fernández-Alonso (1996)), a trait that also is found in other “basal” genera of the /Malvatheca clade sensu Baum et al. (1998), i.e. the clade comprising the /Bombacoideae and /Malvoideae clades). *Phragmotheca* has long apical staminal lobes and five carpels, as does its presumably closest relative *Matisia*. (*Quararibea* has short apical staminal lobes and 2–4 carpels). However, unlike *Matisia*, *Phragmotheca* species also have thicker, prominently ribbed endocarps surrounding the five seeds (cf. fig. 1 in Fernández-Alonso (1996) and Fig. 5E, F).

**Table 1.** Species of *Phragmotheca* Cuatrec. (Malvaceae) known prior to the publication of *P.centinelensis* J.C.Cerón, A.Fernández & J.E.Guevara. Country codes: CO – Colombia, EC – Ecuador, PA – Panama, PE – Peru.

Species	Publication	Distribution	Country	Altitude (m)
*P.siderosa* Cuatrec.*	1946	Chocoan	CO, EC	< 50 (–620)
*P.fuchsii* Cuatrec.	1971	Chocoan	CO	< 50
*P.leucoflora* D.R.Simpson	1982	Amazonian	EC, PE	150–260
*P.amazónica* (W.S.Alverson) Fern.Alonso**	1991	Amazonian	CO, EC, PE	115–150
*P.ecuadorensis* W.S.Alverson	1991	Amazonian	EC	250–450
*P.mammosa* W.S.Alverson	1991	Chocoan	CO, PA	100–800
*P.hydra* Fern.Alonso	1996	Chocoan	CO	50–100
*P.lemniscata* Fern.Alonso	1996	Chocoan	CO	180
*P.rubriflora* Fern.Alonso	1996	Andean	CO	750
*P.siderotricha* Fern.Alonso	1996	Amazonian	PE	320–700
*P.mambitana* Fern.Alonso & R.Jaram.	1999	Cordilleran	CO	1700–1800

* The elevational range of Phragmothecasiderosa includes that of subsp. megacarpa (Fernández-Alonso 1996). **Phragmothecaamazonica was originally published as P.mammosasubsp.amazonica (Alverson 1991).

*Phragmotheca* is a conspicuous floristic element of the humid forests of the Chocó Region of Colombia and Ecuador and the western Amazon. It has been hypothesised that lowland humid forests of Panama, Colombia and Ecuador constitute its centre of diversification (Gentry 1986; Fernández-Alonso 1998). Forests of the Chocó Region of Ecuador are subject to one of the highest deforestation rates in the world. Few remnants persist, accounting for only 15% of the original vegetation cover (Finer and Mamani 2019). Since 2021, we have been carrying out floristic inventories in one of the most iconic and imperilled areas within the Chocó Region of Ecuador: the Centinela (or Montañas de Ila) area. Originally this area had ~ 500 km^2^ of forest, but less than 7 km^2^ remains. In the last 10 years, several new tree species have been described from material collected in Centinela highlighting the relevance of this area for botanical exploration (Torke and Perez 2013; Cornejo 2023; Fernández-Alonso and Cornejo 2024).

During one of our inventories, we collected a specimen of *Phragmotheca* that exhibits remarkable characteristics, clearly different from those of known species in the Chocó (Table 2). This is the first new species of *Phragmotheca* described in the 21^st^ century (Table 1). Thus, our results highlight the importance of botanical exploration and conservation not just in poorly-collected sites in the Neotropics, but specifically in Neotropical cloud-forest remnants.

**Table 2.** Diagnostic characters of *Phragmotheca* Cuatrec. in the Chocó Region of Ecuador and Colombia, including *P.centinelensis* J.C.Cerón, A.Fernández & J.E.Guevara and morphologically similar relatives.

Characters	*P.centinelensis*	*P.hydra*	*P.lemniscata*	*P.rubriflora*	*P.siderosa*
Leaf blade shape	orbicular to oblong-elliptic	cordate-orbicular	broadly elliptic to suborbicular	lanceolate-oblong to narrowly obovate-oblong	ovate to elliptic or broadly elliptic
Abaxial leaf surface indument	sparsely distributed long-branched fasciculate hairs, lepidote-stellate scales and stellate-fasciculate hairs	densely covered by long-branched fasciculate hairs	densely covered by lepidote scales	stellate-lepidote	densely echinate-stellate
Primary leaf veins at base (not counting submarginals)	5	5	3	2	5
Secondary leaf veins (per side)	5	4–5	4–5	6–8	4–5
Calyx tube shape	narrowly campanulate	cylindrical	tubular-fusiform	tubular-cylindrical	tubular
Flower length, including pedicel (cm)	3.3–5.2	2.8–2.9	> 8	6.5–7.0	ca. 9.0
Petal colour	orange or pink	white or yellowish-white	white or yellowish-white	red	white or pale yellowish-brown
Staminal column length, with lobes (cm)	2.9–3.6	1.7	7.0–7.2	6.5	ca. 8.0
Staminal column lobe length (cm)	1.3–1.6	0.9–1.0	1.1–1.3	1.2–1.3	1.5
Thecae per staminal column lobe	6 (relatively uniform in size)	3 (2 large plus 2 basal that are shared with adjacent lobes)	4–6 (quite variable in size)	7 (three pairs per lobe plus one basal that is shared)	–
Number of fovea (septa) per theca	13–14	15–24	few – many (> 50)	many	–
Fruit shape, length and width (cm)	ovoid to broadly ovate, 5.9–7.0 × 4.0–5.3	broadly ovate, 5.2 × 6.0–6.2	spherical, 7.0 × 4.0–4.5	apparently spherical, ca. 4.0–3.8	broadly ellipsoid to sub-obovoid, 6.5–8.0 × 6.3–6.8

## ﻿Methods

After our initial fieldwork during the floristic inventories, we collected additional, fertile specimens of this unknown species of *Phragmotheca*. We also made an exhaustive search of *Phragmotheca* specimens deposited in Ecuador’s three largest herbaria: Herbario de la Universidad Católica del Ecuador (QCA), Herbario Alfredo Paredes de la Universidad Central del Ecuador (QAP) and Herbario Nacional del Ecuador (QCNE; abbreviations according to Thiers (2019)). To gather habit and morphological information, we consulted digitised specimen images of the genus *Phragmotheca* available at the Field Museum virtual herbaria (https://collections-botany.fieldmuseum.org) and 339 images from an archive maintained by one of us [WSA], with additional specimens seen at AMAZ, COL, F, GUAY, HUA, JAUM, MEDEL, MO, MOL, PMA, SCZ, USM and WIS.

To determine the range of the new species for a conservation status assessment using IUCN criteria, we estimated the Extent of Occurrence and Area of Occupancy using the package conR (Dauby et al. 2017). Habitat fragmentation was estimated using the most current deforestation data available for western Ecuador from the online platform Mapa Interactivo (MAATE 2024). We first downloaded ecosystem layers for western Ecuador, as well as deforestation maps for the period 1990–2022. Habitat reduction was then assessed by combining these layers with the layers of Extent of Occurrence (EOO) and Area of Occupancy (AOO) using the clip tool in the ArcGis software (ESRI 2011). The clip tool overlays a range size map of EOO (Extent of Occurrence) and AOO (Area of Occupancy) with boundary layers, which, in this case, include deforestation scenarios from 1990 to 2022 and ecosystem layers. This process refines the potential habitat by limiting it to forest fragments. Habitat loss was then calculated as the area outside the combined boundaries defined by the deforestation and ecosystem layers. Conservation assessment was done following IUCN Standards and Petitions Committee (2022).

## ﻿Results

### ﻿Taxonomic treatment

#### ﻿ Phragmotheca centinelensis

Taxon classificationPlantaeMalvalesMalvaceae

J.C.Cerón, A.Fernández & J.E.Guevara sp. nov.

B7C40E4A-0646-5C97-95AF-9DF1B6BD342C

urn:lsid:ipni.org:names:77359001-1

**Figure 1.**
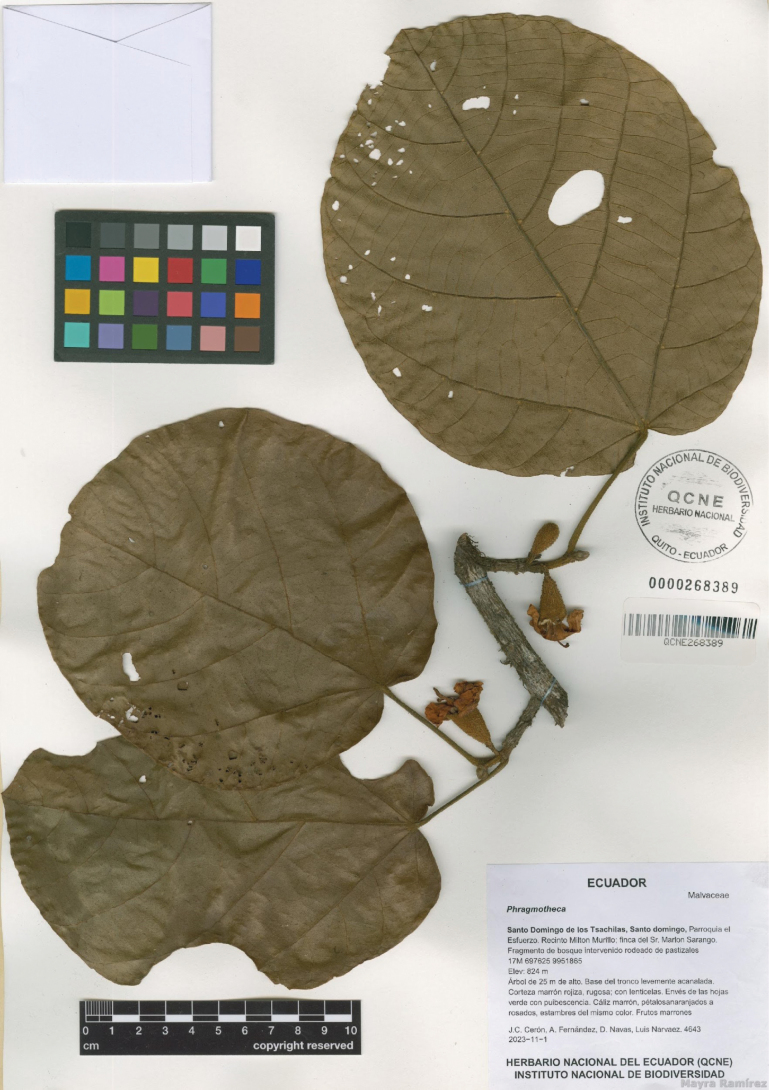
*Phragmothecacentinelensis* J.C.Cerón, A.Fernández & J.E.Guevara, sp. nov. Image of the holotype at QCNE (*J.C. Cerón, A. Fernández, D. Navas, & L. Narváez 4643*).

**Figure 2.**
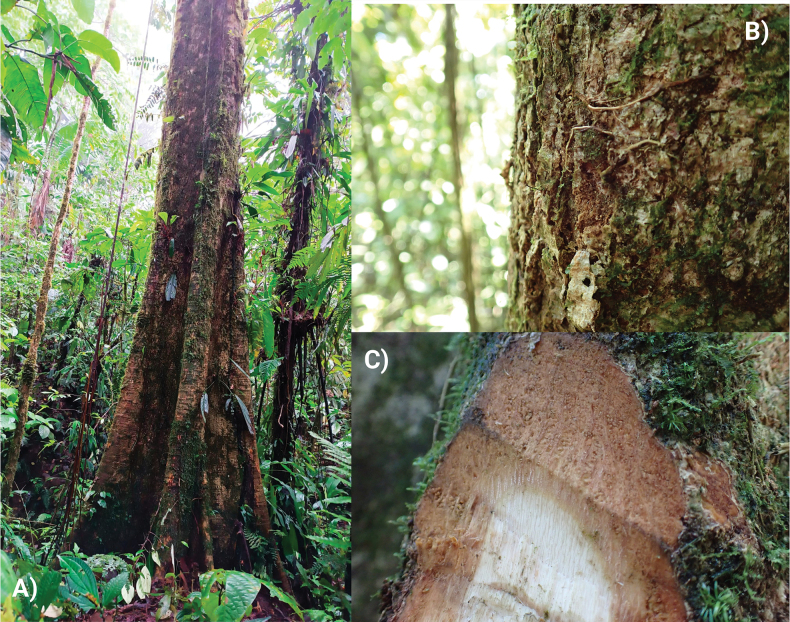
*Phragmothecacentinelensis* J.C.Cerón, A.Fernández & J.E.Guevara **A** trunk **B** outer bark **C** inner bark. Photos of the type individual (*Cerón et al. 4643*) by Andrea Fernández and Juan Carlos Cerón.

**Figure 3.**
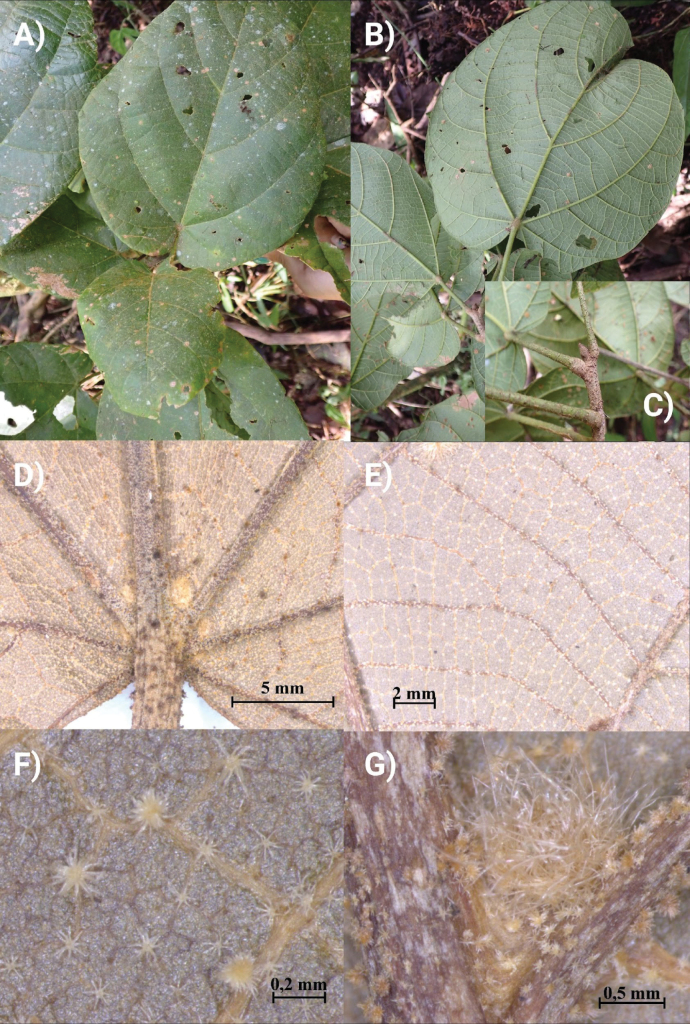
*Phragmothecacentinelensis* J.C.Cerón, A.Fernández & J.E.Guevara **A** leaves, adaxial surface **B** leaves, abaxial surface **C** terminal branch showing the terminal bud and lenticels **D** basal leaf veins, abaxial surface (8×) **E** tertiary and quaternary venation, abaxial surface (8×) **F** stellate-lepidote scales, abaxial surface of leaf (100×) **G** barbate axil of mid-vein and secondary vein, with tufts of long-branched fasciculate hairs and stellate-lepidote scales, abaxial leaf surface. Photos of the type individual (*Cerón et al. 4643*) by Andrea Fernández and Juan Carlos Cerón.

**Figure 4.**
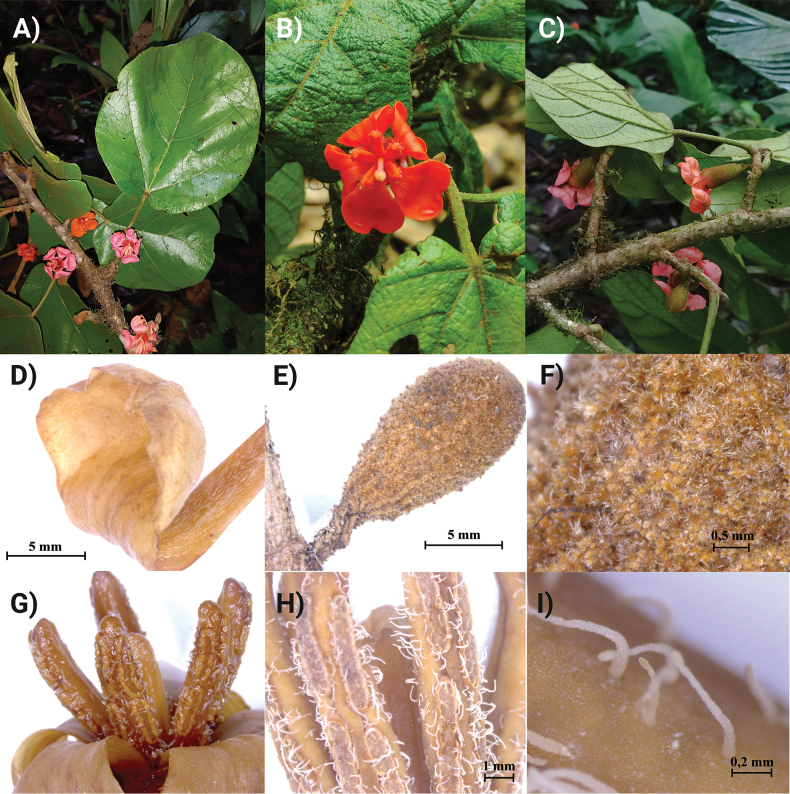
*Phragmothecacentinelensis* J.C.Cerón, A.Fernández & J.E.Guevara **A** flowering branch **B** flower with reflexed, concave-spoon-like petals **C** campanulate flowering calyx **D** distal part of petal showing the diagnostic concave form that is characteristic of this new species **E** flower bud (8×) **F** close-up of flower bud indument, a mix of stellate-lepidote scales and fasciculate hairs **G** apical lobes of staminal column (note sterile tips), each with two parallel rows of thecae and the shorter style in centre **H** close-up of apical lobes with elongate, foveate thecae (15×); **I** elongate, septate hairs on the thecae (100×).

**Figure 5.**
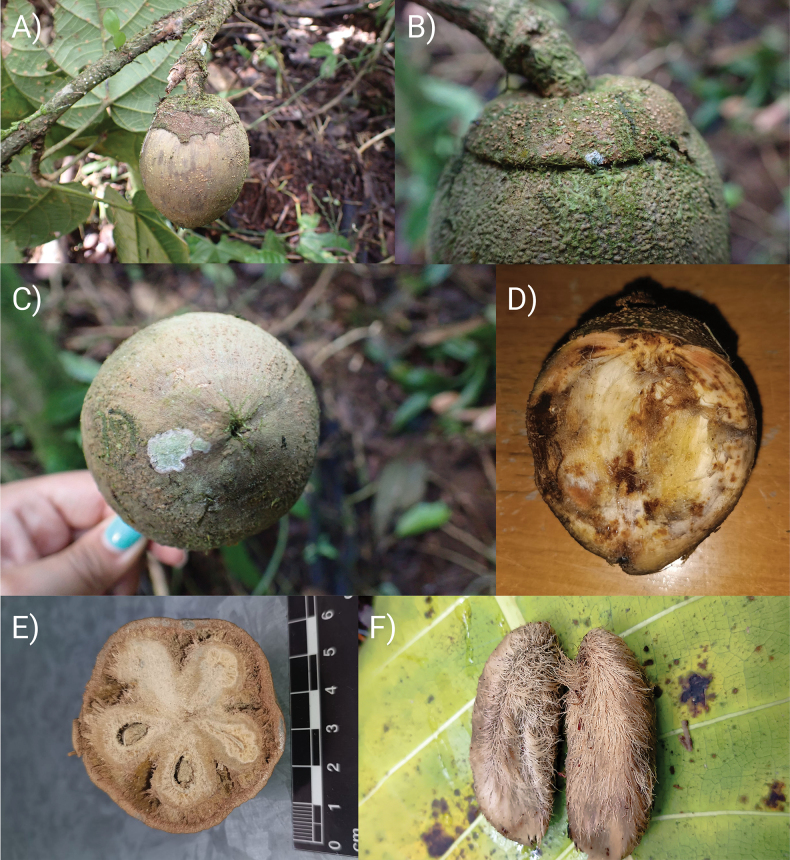
*Phragmothecacentinelensis* J.C.Cerón, A.Fernández & J.E.Guevara **A** fruiting branch **B** patelliform fruiting calyx **C** apex of the fruit showing the fine longitudinal fissures **D** longitudinal view of the fruit showing yellow exudate from the mesocarp **E** transverse section of the fruit showing the exocarp, fibrous mesocarp and very thick endocarp tissue surrounding the seeds **F** two pyrenes (seeds with some residual fibrous-woody endocarp and mesocarp tissue).

Figs 1 , 2 , 3 , 4 , 5

##### Diagnosis.

The new species is morphologically similar to *Phragmothecahydra* Fern.Alonso, but differs by its orbicular to oblong-elliptic (vs. orbicular-cordate) leaves with a mixture of long-branched fasciculate hairs and lepidote-stellate scales in the axils of the mid-vein and secondary veins on the abaxial leaf surface (vs. fasciculate hairs only); larger flowers (3.5–5.0 [including the pedicel] × 2.9–3.5 vs. 2.8–2.9 × 2.8–3.0 cm) with glabrous, concave-spoon-like (vs. linear spathulate) petals; longer, glabrous staminal column (2.9–3.6 vs. 1.7 cm and densely covered by stellate hairs); staminal lobes each bearing 6 thecae (vs. 3–4 thecae); glabrous (vs. sparsely covered with long-branched, fasciculate hairs) style; subcapitate (vs. subacute) stigma; narrower, patelliform fruiting calyx (3.3–3.9 vs. 4–4.5 cm in diameter); and ovoid (vs. globose) fruits that are proportionately more slender (5.0–7.0 × 4.0–5.3 vs. 5.2 × 6.0–6.2 cm in diameter).

The semi-cordate leaf bases of this new species resemble those of *Phragmothecalemniscata* Fern.Alonso, but *P.centinelensis* differs by its larger size (canopy trees to 35 m vs. small treelets); indument of abaxial leaf surfaces (sparse fasciculate hairs, lepidote-stellate scales and stellate-fasciculate hairs vs. dense lepidote scales); floral calyces (narrowly campanulate vs. tubular-fusiform); petals (orange or pink, glabrous, glossy, distally concave, non-reflexed, with slightly convolute margins vs. white-cream, linear-spathulate, distally reflexed, internally covered by a mixture of stellate and lepidote scales); shorter staminal column (2.9–3.6 vs. 7.0–7.2 cm long, including the apical lobes) with apical lobes differing in indument (densely covered by septate simple hairs vs. glabrous); fruiting calyces (patelliform and covered by lenticels vs. discoid sub-cupular); and fruits (broadly ovoid vs. globose).

##### Type.

**Ecuador – Santo Domingo de los Tsáchilas** • (fl, fr, J.C. Cerón, A. Fernández, D. Navas, & L. Narváez 4643 (holotype: QCNE!, isotypes: F!, MO!, QCA!, WIS!)); Parroquia El Esfuerzo, Recinto Milton Murillo, Finca del Sr. Marlon Sarango, fragmento de bosque intervenido rodeado de pastizales; 0°35'54.53"S, 79°13'27.23"W; alt. 824 m; 01 Nov 2023.

##### Description.

***Canopy trees*** to 25–35 m tall; trunk cylindrical, longitudinally fissured and forming thin plates, buttressed to 7 m height (Fig. 2A), outer bark reddish with dispersed, irregular grey spots (Fig. 2B), internal bark fibrous (outer half reddish-brown, inner half yellow-cream; Fig. 2C); ***branches***: main branches off trunk verticillate, smaller branches glabrous, terete, longitudinally fissured, with granular lenticels. ***Stipules*** 2–3 mm long, broadly triangular, caducous. ***Leaves*** alternate, clustered at the tips of the branchlets; ***petioles*** 3.6–12.2 cm long, 1–3.5 mm in diameter, terete, finely striate, densely pubescent, pulvinate at both ends. ***Leaf blades*** (Fig. 3A, B) glaucous when young, pale green abaxially when mature, coriaceous, entire, orbicular to oblong-elliptic, (6–)6.7–31.1(32) × (5–)7.6–29.3(–30) cm, semi-cordate to deeply cordate at base with slightly asymmetric lobes 0.5–4.3 cm depth, the apex obtuse to shortly apiculate; the abaxial surface densely covered by a mixture of lepidote-stellate scales, stellate-fasciculate trichome and fasciculate trichomes; ***venation*** conspicuous on the abaxial surface; primary veins palmate near leaf base (Fig. 3D) with 5 basal nerves, 3–4 submarginal nerves slightly ascending to the leaf margin and with 5 pairs of secondary veins arising from mid-rib in distal 2/3 of blade, these with barbate tufts of fasciculate trichomes and sessile lepidote-stellate scales in their axils (Fig. 3G); tertiary venation also prominent on the abaxial surface (Fig. 3E), forming a conspicuous reticulum (inconspicuous on the adaxial surface), with golden lepidote-stellate scales on the lamina between nerves (Fig. 3F). ***Flowers*** slightly zygomorphic, solitary (Fig. 4A, B), borne opposite to leaves on short branches (brachyblasts), 3.3–5.2 cm long (including the pedicels), the ***pedicels*** 1.2–1.4 cm long, 0.3–0.4 cm in diameter, finely striate, covered with sessile stellate-lepidote scales and fasciculate hairs; ***floral bracts*** absent below the calyx. ***Calyx*** narrowly campanulate (Fig. 4C), 2.3–3.3 × 0.9–1.2 cm, densely covered by a brownish, floccose indument (imparting a granular appearance), broadly acuminate and 4-lobed at summit, internally covered with adpressed sericeous hairs. ***Petals*** spathulate, distinctly bilobed at apex, strongly concave distally (Fig. 4B, D), the apex obtuse or rounded, the margins slightly convolute, 3.5–4.1 × 1.5–2.0 cm, orange or pink, glabrous, glossy, 2–4 inner corolla lobes covering the staminal tube and non-reflexed corolla tube lobes. ***Staminal column*** 2.9–3.6 cm long, the tube (16.5–)16.8–20.6(–21.0) × 0.4–0.6 mm long, orange, with 5 linear terminal lobes (Fig. 4G), each lobe 12.9–15.8 × 2.5–2.8 mm, orange or pink and bearing 6 thecae in two parallel lines on the adaxial surface, these surrounded by long, unbranched, septate hairs (Fig. 4G–I), with 14–16 foveae per theca. ***Ovary*** elongate, 1.5–1.7 mm long, 5-carpellate, glabrous, the style not exceeding staminal filaments in length, longitudinally 6-sulcate, the stigma capitate, spongy, glandular. ***Fruiting pedicel*** short, terete, 7.3–8.9 × 5.8–6.1 mm. ***Fruiting calyx*** patelliform and slightly dentate (Fig. 5A, B), surrounding only the basal 1/5 of the fruit and beset with cream-coloured lenticels. ***Fruit*** ovoid to broadly ovoid, 5.9–7 × 4–5.3 cm, the exocarp pale, smooth, finely fissured longitudinally (Fig. 5C) cream-coloured, sparsely granular with short, fasciculate hairs; the mesocarp fibrous-pulpy, white or cream-coloured and exuding yellowish mucilage (Fig. 5D, E), the endocarp densely fibrous, forming a woody pyrene with each of the five seeds (Fig. 5F). Mature ***seeds*** elliptic, 4–4.8 × 1.7–2 cm.

##### Phenology.

Flowering from November to December; fruiting between February and March.

##### Distribution.

**Figure 6.**
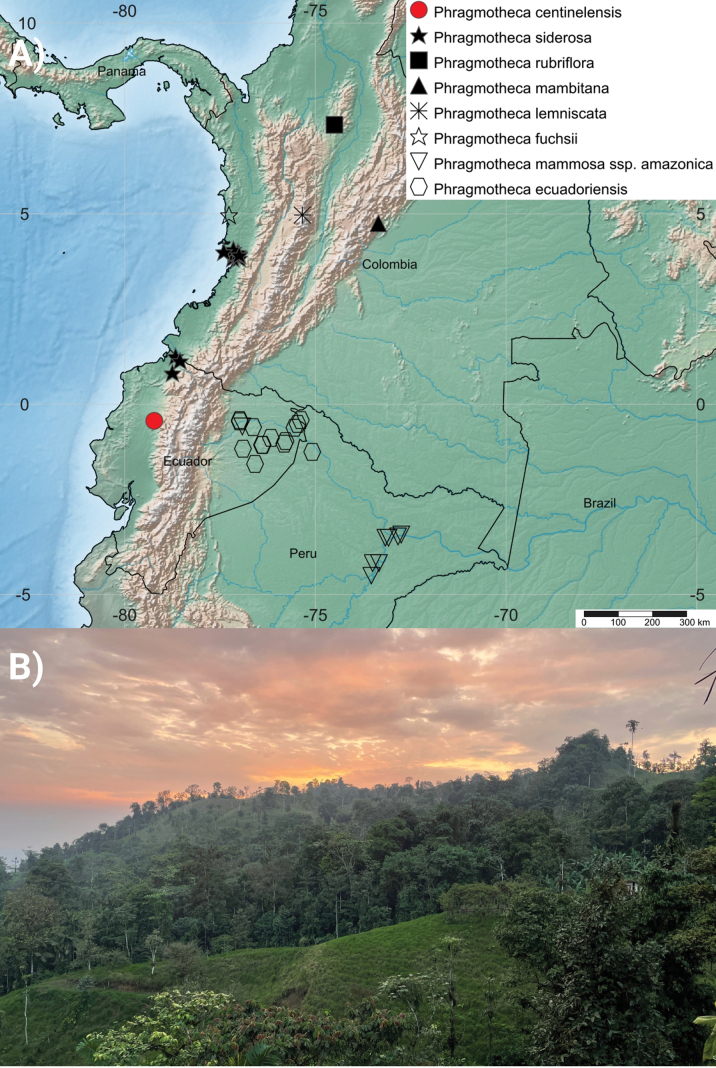
**A** Distribution of *Phragmothecacentinelensis* J.C.Cerón, A.Fernández & J.E.Guevara, its relatives in the Chocó Region of Ecuador and Colombia and two morphologically similar Amazonian species **B** cloud-forest habitat of the new species in Ecuador.

**Figure 7.**
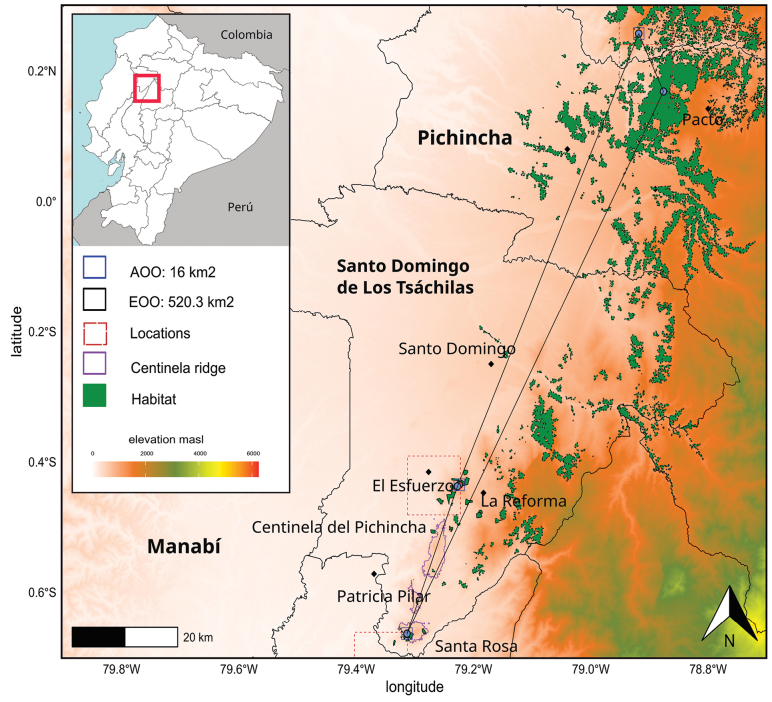
Map showing the Extent of Occurrence (EOO), Area of Occupancy (AOO) and available habitat of *Phragmothecacentinelensis* J.C.Cerón, A.Fernández & J.E.Guevara in western Ecuador. Inset: Location of Santo Domingo de las Tsáchilas Province within Ecuador.

*Phragmothecacentinelensis* is a large tree of non-flooded habitats in the Chocó cloud forests of Ecuador occupying a narrow altitudinal band between 600 and 1000 m elevation. It occupies mostly highly dissected terrain in the ridges of the Centinela area (Figs 6, 7). According to our tree species inventory, *P.centinelensis* appears to be relatively rare in the region and possibly endemic to the Ecuadorian Chocó; the only two known populations are in cloud forest remnants in the western Andean foothills of Centinela. Although we know of no record of *P.centinelensis* from Colombia, it may occur in similar habitats of the adjacent Chocó Department. Despite its rarity, distinctive features make it conspicuous in the field, notably the large buttresses up to 7 m height, its reddish outer bark and the longitudinal fissures on the trunk.

##### Habitat and ecology.

Some of the most conspicuous floristic elements of the Centinela cloud forests include the families Fabaceae, Lecythidaceae and Malvaceae with dominant tree species including *Ocoteainsularis* (Meisn.) Mez, *Ruageaglabra* Triana & Planch., *Guatteria* sp., *Aegiphilaalba* Moldenke, *Trichiliasurinamensis* (Miq.) C.DC., *Socrateaexorrhiza* (Mart.) H.Wendl. and *Iriarteadeltoidea* Ruiz & Pav. The genera *Eschweilera* Mart. ex DC., *Inga* Mill., *Gustavia* L., *Matisia*, and *Quararibea* are also both dominant and species-rich groups in this area. It is remarkable that *Phragmothecacentinelensis* co-exists locally with several other species within the /Matisieae clade, including *M.palenquiana* (A.Robyns) W.S.Alverson, *M.coloradorum* Benoist, *M.giacomettoi* Romero, *M.castano* H.Karst. & Triana and *Q.casasecae* Fern.Alonso & Castrov., *Q.grandifolia* (Little) Cuatrec. perhaps due to similar dispersal mechanisms or pollination syndromes. Natural regeneration of *P.centinelensis* seems to be rare in the forest fragments of the Centinela area. Gravity seems to be the prominent mechanism of primary dispersion. We found a high density of fruits and seeds underneath parent trees with abundant signs of predation by rodents or other small mammals, nearly all fruits with bite marks. There may be secondary dispersal due to scatter-hoarding by these mammals. The heavily armoured endocarps may be an adaptation to facilitate this, but more study is needed. Our current observations suggest a high mortality of seeds and seedlings due to a negative density dependence process via Janzen-Connell phenomena (Forrister et al. 2019).

##### Conservation status.

*Phragmothecacentinelensis* is known from only three confirmed populations in the western foothills of the Ecuadorian Andes in the few remnants of cloud forests of the Montañas de Ila, more specifically in areas outside of Centinela Ridge (Fig. 7). This small population, within a 150-ha patch of forest, represents the type locality. In this area, only three adult individuals have been observed, all of them with a diameter at breast high > 30 cm. Another subpopulation of four individuals has been reported at the southern end of the Centinela Ridge, ca. 15 km from the confirmed population. Based on field observations by other botanists, we believe there to be a second population occurring in a large fragment of forest (2500 ha) in the private Mashpi Reserve, 85 km north of Centinela (A. Pérez and G. Toasa, pers. comm.). We reviewed a sterile *Phragmotheca* herbarium specimen from the Mashpi Reserve and confirmed it belongs to the new species described herein. A third population is located in small patch of cloud forest at 1100–1200 m elevation, near the town of Cielo Verde in Imbabura Province, approximately 15 km north of Mashpi Reserve. This forest is part of the buffer zone from Los Cedros Reserve with approximately 4500 ha protecting the last remnants of Chocó cloud forests in western Ecuador.

Our estimates of extent of occurrence (EOO) and area of occupancy (AOO) are 520 km^2^ and 16 km^2^, respectively (Bachman et al. 2011) (Fig. 7). Two of the three known confirmed populations of *Phragmothecacentinelensis* are not formally protected in the Ecuadorian Protected Area National System (SNAP). Our analysis demonstrates that, from 1990 to 2022, deforestation across the range of this species has reduced its EOO and AOO in 21% approximately. Furthermore, based on estimated EOO and AOO (Fig. 7) and historic deforestation over the last 34 years, we found that 34% of its potential habitat has disappeared. Thus, on the basis of current deforestation rates in the Chocó Region (Finer and Mamani 2019) and habitat loss, our assessment of *P.centinelensis*’s global threat status is Endangered [EN] under IUCN Criterion EN B1ab(i,ii,iii)+2ab(i,ii,iii). It is important to note that historical deforestation of the Centinela Ridge predates the 1970s, but several large forest fragments remained interspersed in a matrix of cacao, balsa and *Gmelina* L. plantations (Dodson and Gentry 1991). However, at present, deforestation was occurring even during our visits: at the time of our floristic inventories in 2021, one of the three adult individuals which we recorded was being removed by loggers.

##### Etymology.

We named the new species in honour of the iconic site that the renowned botanists Alwyn Gentry and Calaway Dodson visited more than 40 years ago in the Centinela area, close to Santo Domingo de los Colorados in the western foothills of the Ecuadorian Andes. At the time they visited an area known as Centinela del Pichincha, they observed a severely fragmented landscape, which led them to conclude that ongoing deforestation had wiped out almost all the remaining forests in the region. After the publication of their seminal paper (Dodson and Gentry 1991), the term “Centinelan extinction” was coined and popularised by Wilson (1992). The term aimed to reflect the global extinction of a high number of endemic plant species, many of them undescribed, following high levels of forest fragmentation.

##### Additional specimens examined.

**Ecuador – Santo Domingo de los Tsáchilas** • J.C. Cerón et al. 4684 (QCA, QCNE, F); Parroquia El Esfuerzo, Recinto Milton Murillo, Finca del sr. Marlon Sarango. bosque siempreverde piemontano de la cordillera occidental de los Andes (BsPn01), Fragmento de bosque intervenido rodeado de pastizales; 0°26'15.35"S, 79°13'38.57"W; alt. 840 m; 01 Nov 2023; fl • **Santo Domingo de los Tsáchilas**: J.C. Cerón et al. 4688 (QCA, QCNE, F); Parroquia Centinela del Pichincha, Comunidad Santa Rosa, bosque siempreverde piemontano de la cordillera occidental de los Andes (BsPn01), bosque maduro intervenido; 0°39'51.15"S, 79°18'48.37"W; alt. 682 m; 01 Nov 2023; fr. • **Pichincha**: Reserva Masphi • PDRBA 43 (QCA); Colecciones en la parcela 7 de 60 × 60 m del transecto altitudinal para el monitoreo de la dinámica forestal. Bosque Montano Bajo. Árbol, placa 7294; 0°10'6.63"N, 78°52'34.2402"W; alt. 800–900 m; 2–25 Aug 2019. • **Imbabura**: Cielo Verde • PDRBA 285 (QCA); Colecciones en la parcela 7 de 60 × 60 m del transecto altitudinal para el monitoreo de la dinámica forestal. Bosque Montano Bajo. Árbol, placa 586; 0°15'26.2074"N, 78°55'6.096"W; alt. 1100–1200 m; 10–22 Jul 2021.

## ﻿Discussion

Based on morphology and geographic distribution, *Phragmothecacentinelensis* is a new species distinct from other morphologically similar congeneric species in the Chocó and Amazon Regions.

*Phragmothecacentinelensis* is one of many new species described from remnant forests in western Ecuador over the last 15 years (Cornejo 2009a, 2009b,; Palacios 2012; Torke and Perez 2013; Guevara-Andino and Fernández-Fernández 2020; Couvreur et al. 2022; Fernández-Alonso and Cornejo 2024; Clark et al. 2024). Its discovery adds valuable new insight into the region’s conservation value and floristic richness, as well as providing another piece of the puzzle to understand the historical biogeography and evolution of members of the /Matisieae clade (the > 100 species included in the genera *Matisia*, *Phragmotheca* and *Quararibea*). For example, *M.palenquiana* (A.Robyns) W.S.Alverson was described (as a *Quararibea*) from specimens collected in 1974 by Gentry and Dodson at the Río Palenque Biological Station, very close (60 km) to the type locality of *P.centinelensis*. The diagnosis in the original article (Robyns 1976) notes the similarity of the new species to *M.bicolor* Ducke, an Amazonian species. A third, very closely-related species, with similarly distinctive flowers and fruits, was recently discovered in Costa Rica notwithstanding many decades of dedicated botanical collecting efforts there: *M.tinamastiana* A. Estrada & Cascante (Estrada and Cascante (1998), with an updated distribution in Fernández-Alonso and Campos-Pineda (2023)). Triplets of closely-related species distributed east of the Andes in the Amazon, west of the Andes in Colombia and Ecuador and northwest into isthmian Costa Rica and Panama are evident in several genera of the /Bombacoideae and /Malvoideae clades (sensu Baum et al. (1998, 2004)), which invites further investigation once additional molecular phylogenies for these genera are available.

## ﻿Conclusion

*Phragmothecacentinelensis* is a distinct and endangered new species of canopy tree found in forest remnants of western Ecuador. Discovery and documentation efforts for rare species such as this are critical to their conservation and to the value they add to our understanding of floristics, historical biogeography and the evolutionary diversification of Neotropical cloud forests.

## Supplementary Material

XML Treatment for Phragmotheca centinelensis

## ﻿Additional information

### Author ORCIDs

Juan Ernesto Guevara-Andino https://orcid.org/0000-0002-5433-6218

Dawson M. White https://orcid.org/0000-0002-0670-9390

Nigel C. A. Pitman https://orcid.org/0000-0002-9211-2880

Juan-Carlos Cerón https://orcid.org/0009-0007-4986-4802

Andrea Fernández https://orcid.org/0009-0002-0445-2867

Daniel Navas-Muñoz https://orcid.org/0009-0001-1761-9147

William S. Alverson https://orcid.org/0000-0001-7793-0386

### Data availability

All of the data that support the findings of this study are available in the main text or Supplementary Information. Coding for the IUCN analyses and distributional maps will be submitted to Dryad.

## ﻿Appendix 1

This appendix considers the option of placing the genus *Phragmotheca* Cuatrec. and two closely-related genera in the /Matisieae clade (*Matisia* and *Quararibea*, Baum et al. (2004)), in the Matisieae within the Malvoideae, versus placing it in the newly-published Matisioideae (Colli-Silva et al. 2025). We use forward slashes (“clademarks,” as described in Baum et al. (1998)) for the names of clades, to avoid confusion with ranked names, which are, in many cases, otherwise identical.

Recent phylogenetic studies consistently indicate a clade comprising one branch with all members of the traditional family Malvaceae and a second branch with most members of the traditional family Bombacaceae (excluding *Durio* Adans. and relatives). This clade was given the name /Malvatheca (fig. 2, in Baum et al. (2004)). Its two major branches, the /Malvoideae and /Bombacoideae clades, were given stem-based phylogenetic definitions (appendix in Baum et al. (1998)). Ranked names for these two clades, Malvoideae and Bombacoideae, came into general use by researchers in the last two decades and in authoritative references such as Stevens (2001 onwards). Colli-Silva et al. (2025) acknowledge the close phylogenetic relationship of these two major lineages, but they constrain the application of Malvoideae to the distal portion of the /Malvoideae clade, which then necessitates recognition of the /Matisieae clade as a subfamily, Matisioideae. However, this narrowed application of the ranked name Malvoideae has consequences that may not be desirable.

Under their proposal, the /Malvoideae clade would no longer have a ranked name available for it, despite being a lineage with a complex and interesting pattern of floral, fruit and biogeographic evolution and despite its sister lineage retaining its subfamilial rank: Colli-Silva et al. (2025) did not elevate the three currently recognised tribes of Bombacoideae (Adansonieae Horan, Bernoullieae Carv.-Sobr. and Bombaceae Kunth.; Carvalho-Sobrinho et al. (2016)) to subfamilies, notwithstanding molecular and morphological support comparable to their Matisioideae and Malvoideae.

The second set of consequences may obtain when better phylogenetic trees are available for the base of the /Malvatheca clade. For example, the genera *Ochroma* Sw. (1 sp.) and *Patinoa* Cuatrec. (4 spp.) have been recovered in some phylogenetic studies (Alverson et al. 1999; Baum et al. 2004; Carvalho-Sobrinho et al. 2016) as basal branches of the /Malvoideae clade (a quite plausible result, given aspects of their morphology) and phylogenetic placements of the genera *Chiranthodendron* Larreat. (1 sp.), *Fremontodendron* Coville (3 spp.) and *Septotheca* Ulbr. (1 sp.) are still unclear. With the exception of *Fremontodendron*, no representatives of these genera were included in the study by Colli-Silva et al. (2025). If any of these are found to be basal branches of the /Malvoideae clade, then they will require one or more new subfamily names despite comprising only one or a few species. In contrast, the broader concept of Malvoideae that has been in general use over the last two decades would not require the designation of additional, tiny subfamilies. Alternatively, assigning the rank of supertribe (Ezedin 2024) to the segment of the malvoid lineage that has echinate pollen (i.e. the Malvoideae of Colli-Silva et al. (2025)) would also avoid these problems. If, in the future, more comprehensive phylogenetic studies find that the /Matisieae clade is in a polytomy with the /Bombacoideae and /Malvoideae clades or attaches outside of the /Malvatheca clade, it would make sense to elevate the current tribe, Matisieae, to subfamilial status. However, that is not our current understanding of relationships.

Thus, narrowing the definition of Malvoideae and elevating the Matisieae to subfamilial status seems premature or even unwarranted and does not add significant insight into the evolution of these groups. Assignment of ranks is arbitrary, but requires adequate justification, especially since changing them can create confusion through creating multiple meanings of the same name(s). Therefore, until convincing phylogenetic studies are available that include representatives of at least *Ochroma*, *Patinoa* and *Septotheca*, we prefer to retain the concepts of Malvoideae and Matisieae that have been in general use during the last two decades.

## Supplementary materials

Supplementary material 1 Conservation assessment and georeferenced data https://doi.org/10.3897/phytokeys.254.143106.suppl1 Data type xlsx Explanation note This file contains information regarding herbarium specimens and habitat reduction analyses used for the description of the new tree species *Phragmothecacentinelensis*. The different spreadsheets contains georeferenced data, collectors' name, herbaria names and data on habitat reduction, deforestation, extent of occurrence and area of occupancy. https://binary.pensoft.net/file/1287969This dataset is made available under the Open Database License (http://opendatacommons.org/licenses/odbl/1.0/). The Open Database License (ODbL) is a license agreement intended to allow users to freely share, modify, and use this Dataset while maintaining this same freedom for others, provided that the original source and author(s) are credited.Juan Ernesto Guevara-Andino, Dawson M. White, Nigel C. A. Pitman, Juan-Carlos Cerón, Andrea Fernández, Daniel Navas-Muñoz, William S. Alverson

## References

[B1] Alverson WS (1991) A synopsis of *Phragmotheca* (Bombacaceae), with two new species and a new subspecies. Brittonia 43(2): 73–87. 10.2307/2807297

[B2] Alverson WS Whitlock BA Nyffeler R Bayer C Baum DA (1999) Phylogeny of the core Malvales: Evidence from *ndh*F sequence data. American Journal of Botany 86(10): 1474–1486. 10.2307/2656928 10523287

[B3] Bachman S Moat J Hill A de la Torre J Scott B (2011) Supporting Red List threat assessments with GeoCAT: Geospatial conservation assessment tool. ZooKeys 150: 117–126. 10.3897/zookeys.150.2109 PMC3234434 22207809

[B4] Baum DA Alverson WS Nyffler R (1998) A durian by any other name: Taxonomy and nomenclature of the Core Malvales. Harvard Papers in Botany 3(2): 315–330.

[B5] Baum DA DeWitt Smith S Yen A Alverson WS Nyffeler R Whitlock BA Oldham RL (2004) Phylogenetic relationships of Malvatheca (Bombacoideae and Malvoideae; Malvaceae sensu lato) as inferred from plastid DNA sequences. American Journal of Botany 91(11): 1863–1871. 10.3732/ajb.91.11.1863 21652333

[B6] Carvalho-Sobrinho JG Alverson WS Alcantara S Queiroz LP Mota AC Baum DA (2016) Revisiting the phylogeny of Bombacoideae (Malvaceae): Novel relationships, morphologically cohesive clades, and a new tribal classification base on multilocus phylogenetic analyses. Molecular Phylogenetics and Evolution 101(2016): 56–74. 10.1016/j.ympev.2016.05.006 27154210

[B7] Clark JL Fernández A Zapata JN Restrepo-Villarroel C White DM Pitman N (2024) *Amalophyllonmiraculum* (Gesneriaceae), an exceptionally small lithophilous new species from the western Andean slopes of Ecuador. PhytoKeys 242: 307–316. 10.3897/phytokeys.242.118069 38903848 PMC11188077

[B8] Colli-Silva M Pérez-Escobar OA Ferreira CDM Costa MTR Gerace S Coutinho TS Yoshikawa VN Antonio-Domingues H Hernández-Gutiérrez R Bovini MG Duarte MC Cheek M Chase MW Fay MF Christenhusz MJM Dorr LJ Schoeplein C Cocoran M Roy S Cable S McLay T Maurin O Forest F Baker WJ Antonelli A (2025) Taxonomy in the light of incongruence: An updated classification of Malvales and Malvaceae based on phylogenomic data. Taxon, Early View [published 23 Jan 2025]. 10.1002/tax.13300

[B9] Cornejo X (2009a) Two new species of *Pentagonia* (Rubiaceae, Hippotideae) from Colombia and Ecuador. Novon 19(1): 25–31. 10.3417/2006060

[B10] Cornejo X (2009b) *Amyriscentinelensis* and *Zanthoxylumbonifaziae*: Two new species of Rutaceae from western Ecuador. Harvard Papers in Botany 14(2): 161–166. 10.3100/025.014.0207

[B11] Cornejo X (2023) *Eschweilerapodoaquilae*: A new species of Lecythidaceae from northwestern Ecuador. Phytotaxa 579(2): 139–142. 10.11646/phytotaxa.579.2.8

[B12] Couvreur TLP Cornejo X Zapata JN Loor A (2022) Two new magnoliid (Annonaceae, Lauraceae) tree species from Manabí, western Ecuador. Blumea 67(2): 97–108. 10.3767/blumea.2022.67.02.02

[B13] Dauby G Stévart T Droissart V Cosiaux A Deblauwe V Simo-Droissart M Sosef MSM Lowry PP 2nd Schatz GE Gereau RE Couvreur TLP (2017) *ConR*: An R package to assist large-scale multispecies preliminary conservation assessments using distribution data. Ecology and Evolution 7(24): 11292–11303. 10.1002/ece3.3704 29299301 PMC5743656

[B14] Dodson CH Gentry AH (1991) Biological extinction in western Ecuador. Annals of the Missouri Botanical Garden 78(2): 273–295. 10.2307/2399563

[B15] ESRI (2011) ArcGIS Desktop, Release 10. Environmental Systems Research Institute, Redlands, California.

[B16] Estrada A Cascante A (1998) *MatisiaTinamastiana* (Bombacaceae): Una nueva especie arborescente del Pacífico Central de Costa Rica. Brenesia 49–50: 79–85. https://biblioteca.museocostarica.go.cr/articulo.aspx?id=2519&art=9154

[B17] Ezedin Z (2024) A conspectus of Angiosperm supertribes. Harvard Papers in Botany 29(1): 63–78. 10.3100/hpib.v29iss1.2024.n8

[B18] Fernández-Alonso JL (1996) Contribuciones al conocimiento del género *Phragmotheca* Cuatr. (Bombacaceae-Quararibeae). Caldasia 18(3): 253–284. https://repositorio.unal.edu.co/handle/unal/31015

[B19] Fernández-Alonso JL (1998) Redescripción del “Piscande”, *Pachirapatinoi* (Dugand & Robyns) Fern.-Alonso comb. nov. (Bombacaceae) y notas sobre su hábitat y distribución. Revista de la Academia Colombiana de Ciencias Exactas 22(82): 7–12. 10.18257/raccefyn.22(82).1998.2874

[B20] Fernández-Alonso JL Campos-Pineda E (2023) *Matisiagentryi* and *M.tinamastiana* (Malvaceae), two species newly recorded from Panama and an updated key to *Matisia* in this country. Checklist 19(6): 1013–1020. 10.15560/19.6.1013

[B21] Fernández-Alonso JL Cornejo X (2024) *Quararibeacentinelae* (Malvaceae), una nueva especie endémica de Centinela, occidente de Ecuador. Harvard Papers in Botany 29(1): 79–85. 10.3100/hpib.v29iss1.2024.n9

[B22] Fernández-Alonso JL Jaramillo-Mejía R (1999) Nueva especie de zapote del monte (*Phragmotheca*, Bombacaceae) en bosques premontanos del centro de Colombia. Caldasia 21(2): 125–131. https://repositorio.unal.edu.co/bitstream/handle/unal/31131/17513-55778-1-PB.pdf?sequence=1

[B23] Fernández-Alonso JL Fernández-Hilario R Reynel-Rodríguez C (2017) Redescripción del zapotillo rosado del Perú, *Phragmothecasidereotricha* Fern. Alonso (Malvaceae) y notas sobre su hábitat y distribución. Revista de la Academia Colombiana de Ciencias Exactas, Físicas y Naturales 41(160): 319–325. 10.18257/raccefyn.498

[B24] Finer M Mamani N (2019) Saving the Ecuadorian Chocó. MAAP: 102. https://www.maapprogram.org/choco/

[B25] Forrister DL Endara MJ Younkin GC Coley PD Kursar TA (2019) Herbivores as drivers of negative density dependence in tropical forest saplings. Science 363(6432): 1213–1216. 10.1126/science.aau9460 30872524

[B26] Gentry A (1986) Species richness and floristic composition of Choco region plant communities. Caldasia 15(71–75): 71–91. https://www.jstor.org/stable/43406071

[B27] Guevara Andino JE Fernández-Fernández D (2020) A new rare and endemic species of *Sloanea* (Elaeocarpaceae) from the Chocó region of Ecuador. PhytoKeys 160: 131–139. 10.3897/phytokeys.160.54993 32982554 PMC7492195

[B28] IUCN Standards and Petitions Committee (2022) Guidelines for Using the IUCN Red List Categories and Criteria. Version 15.1.

[B29] MAATE: Ministerio del Ambiente, Agua y Transición Ecológica del Ecuador (2024) Mapa interactivo. http://ide.ambiente.gob.ec/mapainteractivo/ [accessed 14 Nov 2024]

[B30] Palacios W (2012) Cuatro especies nuevas de árboles del Ecuador. Caldasia 34(1): 75–85. https://revistas.unal.edu.co/index.php/cal/article/view/36428

[B31] Reveal JL (2012) Matisieae and Matisiinae. Indices Nominum Supragenericorum Plantarum Vascularium. http://www.plantsystematics.org/reveal/pbio/fam/allspgfileM.html [accessed 14 Nov 2024]

[B32] Robyns A (1976) Bombacaceae neotropicae novae VII: A new species of *Quararibea* from Ecuador. Bulletin du Jardin Botanique National de Belgique 46(1/2): 235–236. 10.2307/3667417

[B33] Schumann K (1895) Matisieae. In: Engler A, Prantl K (Eds) Die Natürlichen Pflanzenfamilien III, 6: 58, 63–65. https://www.biodiversitylibrary.org/item/56529

[B34] Stevens PF (2001 onwards) Angiosperm Phylogeny Website, version 17, updated 4 Oct 2024. Missouri Botanical Garden, St. Louis. https://www.mobot.org/MOBOT/research/APweb/

[B35] Thiers B (2019) Index Herbariorum: A global directory of public herbaria and associated staff. New York Botanical Garden, New York. https://sweetgum.nybg.org/science/ih/

[B36] Torke B Perez A (2013) Notes on the genus *Swartzia* (Leguminosae) in Ecuador, with descriptions of two new species. Phytotaxa 147: 13–25. 10.11646/phytotaxa.147.1.2

[B37] Wilson EO (1992) The Diversity of Life. Belknap Press, Cambridge.

